# Exploring Critical Components of Physician-Patient Communication: A Qualitative Study of Lay and Professional Perspectives

**DOI:** 10.3390/healthcare11020162

**Published:** 2023-01-05

**Authors:** Nurul Ain Mohd Salim, Nurhanis Syazni Roslan, Rafidah Hod, Syahiera Farhana Zakaria, Siti Khadijah Adam

**Affiliations:** 1 Department of Human Anatomy, Faculty of Medicine and Health Sciences, Universiti Putra Malaysia, UPM, Serdang 43400, Malaysia; 2 Department of Medical Education, School of Medical Sciences, Universiti Sains Malaysia, Kota Bharu 16150, Malaysia; 3 Medical Education Research and Innovation Unit (MERIU), Faculty of Medicine and Health Sciences, Universiti Putra Malaysia, UPM, Serdang 43400, Malaysia; 4 Department of Pharmacy Practice, School of Pharmacy, International Medical University, Bukit Jalil, Kuala Lumpur 57000, Malaysia

**Keywords:** communication skill, physician-patient communication, physician-patient relationship

## Abstract

(1) Background: The ability to communicate with patients and their relatives is a crucial skill for a physician. Unfortunately, many physicians and medical students are not well-equipped in this area. Therefore, this study aims to better understand the views on critical components of physician-patient communication to improve their skills. (2) Methods: This qualitative study utilized focus group discussions (FGDs) and in-depth interviews (IDIs). Through a purposive sampling technique, 32 medical students and physicians from the Faculty of Medicine and Health Sciences, Universiti Putra Malaysia (FMHS UPM) and Universiti Putra Malaysia Teaching Hospital (HPUPM), as well as patients and relatives from government and private hospitals or clinics were recruited. All sessions were audio-recorded, transcribed, and analyzed thematically. (3) Results: Seven themes were identified: professionalism, content of communication, verbal, non-verbal and paraverbal communication skills, environment, and visual communication. Good eye contact, providing treatment plans, and ensuring patient privacy and confidentiality were emphasized by physicians and medical students. In comparison, patients and relatives focused on the prognosis of disease, physician’s empathy and advice, and physician’s skills in building rapport with their patients and relatives. (4) Conclusion: The critical components that were highlighted by both professionals and laymen in the study should be practiced to ensure effective communication between physician and patient. There were different expectations in terms of the content of information between both groups. Patients and relatives were more interested in the physician’s advice regarding their diet, care plans, physical activities, and daily routine. They were also focused on the prognosis of the disease, which indicates how quickly they would get better. Meanwhile, physicians and medical students were concentrating on management and treatment strategies, such as what additional procedures should be considered and what medications might work best for their patients. We also found that the patients and relatives had a lack of awareness on confidentiality issues. These findings provide an insight on the improvement of medical training and patient education to improve patient care. Patients have a right to privacy protection, and physicians should be well trained to carry out all procedures and treatment plans to ensure patients are treated with respect.

## 1. Introduction

Good physician-patient communication is among the essential components of the healthcare system. A physician’s communication skills comprise the ability to gather information to facilitate an accurate diagnosis, counsel appropriately, give therapeutic instructions, and establish caring relationships with patients [1,2]. Good physician-patient communication may support patient health due to increased comprehension, stress reduction and increased compliance. Hence, patients would be more inclined to concede health problems, understand their treatment options, modify their behavior accordingly and comply with their medications [3,4]. Good physician-patient communication is an essential building block for informed consent and decision-making by the patient.

Non-verbal communication skills, verbal communication skills, content of communication, and attitudes were found to be important components in physician-patient interaction [5]. A study by Ranjan et al. [6] and Bone et al. [7] mentioned that for a physician to maintain good communication with the patient, he or she needs to have good verbal communication skills such as listening skills, greetings their patients, and thanking them for their cooperation. Being more empathetic and sympathetic are two important behaviors that physicians should express when communicating with patients, since these qualities directly impact how satisfied the patient is [6,8,9]. Furthermore, another study showed that a physician should always convey sufficient information to patients, besides building trust with their patients to clear any misunderstandings [10]. Unfortunately, those skills are, in many cases, neglected by today’s physicians [11]. Additionally, physicians from a previous study felt that patient education may facilitate effective decision-making. Patients, on the other hand, focused on the physician’s opinion and tried to understand the possible treatment options [12]. Another study found that physicians who explicitly expressed doubt in the diagnosis, and who lacked sufficient explanation due to insufficient medical competency, led to lower levels of trust and confidence as well as reduced adherence to treatment [13].

Malaysia has a well-established and efficient health sector, with many public and private healthcare providers. Good communication skills in healthcare can facilitate and enrich face-to-face consultations between physicians and patients. Unfortunately, physicians continue to demonstrate poor physician-patient communication skills, even though communication training is provided in medical schools. A study also mentioned that knowledge of physician-patient communication skills in Malaysia is still in its infancy among medical students [13]. Incompetency in communication by healthcare providers often leads to dissatisfaction and poor patient outcomes [14,15].

A study conducted by Vimala et al. (2016) showed that several circumstances contributed to inefficient communication, such as during interactions when there is a language barrier and different cultural backgrounds. The study also mentioned that non-verbal indicators such as poor eye contact, judgmental facial expression, harsh or angry tones, inappropriate body language and visual gestures may hinder the physician-patient relationship [3]. Therefore, physicians need to have the necessary training to overcome any communication hurdles that may arise [16].

Schramm’s Model of Communication is one of the popular models in communication, including physician-patient communication. There are six components of communication proposed by this model; namely, the source, encoder, message, channel, decoder, and receiver. This model encompasses verbal, non-verbal and paraverbal communication. It also emphasizes that the sender, who can be either a physician or a patient, can deliver the information via a variety of methods such as by using simple words, models, and soft tone of voice to establish an appropriate two-way communication [17,18].

It is also important to note that culture plays an important role in physician-patient communication. A previous study by Claramita et al. [19] discovered large differences in physician-patient communication between Southeast Asia and Western countries. For example, patient information such as sexual activity, drug, alcohol intake, and mental health are more sensitive in Southeast Asia compared to Western countries. Consequently, certain physicians in Malaysia tend to lack the skills to communicate with patients involving the above issues [20], leading to ineffective communication between physicians and patients, and missing patient information that could impact diagnostics as well as therapy. Therefore, this study aims to explore the critical components of communication in the physician-patient relationship that tend to be neglected by physicians. The findings of this study can contribute to the enhancement of communication skills training in medical schools, leading to improved patient care and eventually an improved healthcare setting.

## 2. Materials and Methods

### 2.1. Design

A qualitative design was applied to gain a thorough knowledge of the critical components of communication from the perspectives of professionals (namely, physicians) and medical students, and from laymen who have experienced being a patient or a relative to a patient. Phenomenology research design was adopted in this study as the authors attempted to understand and describe professional and laymen viewpoints on important communication elements in the context of clinical and academic settings. Focus group discussions (FGDs) were conducted with physicians and medical students. In-depth interviews (IDIs) were done with patients and relatives to facilitate their participation and to make it easier for them to convey their opinions. The authors attempted to construct the reality by understanding in-depth the experiences of physicians, trainees, patients and relatives regarding the critical components of physician-patient communication in healthcare and academic settings. The authors interacted with the participants and put themselves into the scenario. They used qualitative interpretive methods with a well described context, and conducted FGDs and IDIs to collect the data.

### 2.2. Participants

Physicians and medical students in this study were recruited from the Universiti Putra Malaysia Teaching Hospital (HPUPM) and the Faculty of Medicine and Health Sciences (FMHS). Patients and relatives from both public and private hospitals or clinics were recruited. The first author obtained a list of potential participants for professional groups from HPUPM and FMHS UPM. The medical students were subdivided into academic years. By using this list, participants for each group were selected through the stratified purposive sampling technique. Additionally, because the first author recruited participants without having a list of potential participants, they used criterion-based purposive sampling to select individuals from laymen who had experience with physician-patient communication. Moreover, the authors employed maximum variation sampling to ensure that the unit of analysis reflects a diverse group in terms of gender and ethnicity in Malaysia. It might involve a variety of perspectives and experiences due to differences in gender and ethnicities. Male and female, as well as Malay, Chinese, and Indian participants were invited for the study. The inclusion criteria included Malaysians, so that the collected data are aligned with a Malaysian context. As the first-year medical students may have lack of adequate training in communication skills, only second to fifth year students were invited for the study. The physicians and medical students are considered professionals because they are formally trained in physician-patient communication at medical school. Even though physicians may forget what they have learned previously, they are able to retain and apply the skills in their daily service and their experience after years in service. On the other hand, the laymen (namely, the patients and relatives in this study) have not been formally trained in physician-patient relationships, but they have various experience interacting with physicians during their hospital or clinic visits.

### 2.3. Data Collection

The FGDs and IDIs were guided by a semi-structured interview that was created based on the literature. The sessions began with the first author, acting as the moderator of the session, asked an open-ended question, such as *“Based on your experience, what is the essential element when communicating with your physician/patient/relative?”* Based on the participants’ responses, the author then began asking more specific questions, such as *“In your experience, how well did your physician/patient interact verbally with you?”* or *“Can you suggest any important elements to ensure that your communication becomes more effective?”* Moreover, the moderator would probe those who tended to go silent, and attempted to refocus the participant’s attention when they deviated from the research topic. One FGD served as the pilot, and the guide was amended based on the feedback from the research team. The sessions were performed online using Google Meet or Zoom platforms.

Physicians and medical students spent an average of 70 and 72 min during the FGDs, which lasted approximately 50 to 90 min, respectively. The length of the IDIs ranged from 20 to 50 min, with patients spending an average of 35 min and relatives taking an average of 37 min. All sessions were audio recorded, while the transcriptions were conducted verbatim. The sessions were carried out by the first author between March 2021 and March 2022. Data collection continued until data saturation was reached. After four FGD sessions with medical students and physicians, we achieved data saturation in our study. For IDIs, the data saturation point was achieved after the fifth and sixth interviews with patients and relatives, respectively. Saturation is reached when individuals consistently repeat the same data and when no new information, such as personal insights or experiences, are provided by the participants. For instance, when participants repeatedly state that using the preferred language with simple words and listening attentively are critical components of verbal communication skills, with no additional information provided, data saturation has been reached.

### 2.4. Data Analysis

Themes, several levels of categories, and initial coding were all part of the data analysis process. The data were managed using Atlas.ti software version 9, and a thematic map was constructed in the software to see the relationship between themes. The authors followed Braun and Clarke’s six-phase structure (2006) to analyze the data. Prior to continuing, the authors must read and re-read all the transcripts to familiarize themselves with the entire body of data before they go any further. For the second step, before determining and finalizing the themes, all transcripts were coded to reduce lots of data into small chunks of meaning. The open-ended questions that were asked throughout the sessions, and the purpose of the study were used as a guideline to generate the codes, known as open-coding. Subsequently, the codes were then examined, and those that fit together were organized into categories and themes. In the fourth step, the authors reviewed all codes and categories to ensure all codes were grouped into suitable categories, and those categories were matched into the correct themes. The authors then had to define each theme in order to identify its “essence” and the ways in which the themes interacted with one another, and report the findings of their thematic analysis [21].

### 2.5. Trusworthiness of the Study

The first author was trained in conducting FGDs, IDIs, and qualitative data analysis in order to ensure credibility. The dependability of the analysis was ensured by having the authors and participants review each transcript for transcribing accuracy. Each transcript was read by the first author several times to become familiar with the general patterns in the participants’ responses. The author then developed codes from each transcript, and these codes were verified by conducting code-recoding and comparing the new codes to the old codes. An external auditor with experience in qualitative data analysis was hired by the authors. The thematic analysis data, which comprised quotations, codes, categories, and themes, were reviewed by the expert. In certain cases, the first author sought clarification from the participants to guarantee conformability and prevent misinterpretation of data. Since the findings were mutually exclusive and directly addressed the research question, the authors ensured that those professional and lay perspectives and experiences described provided valuable information that can be used for future study related to the physician-patient communication in various contexts. Moreover, the authors created an audit trail by recording and documenting all research-related activities that were supported by evidence, such as details on FGDs and IDIs sessions, and the participant’s profiles.

### 2.6. Researchers’ Characteristics and Reflexivity

N.A.M.S is a postgraduate student at UPM, and is the first author and researcher that conducted FGDs and IDIs. The author does not work either as a physician or a lecturer at both FMHS and HPUPM, therefore, the author had no authority and did not hold any positions that might risk the participants. The author also does not mislead the interpretation of the data as she is not familiar with the participants.

R.H and S.K.A both hold academic positions at FMHS, and are familiar with the medical programs that have been taught there. They could interpret the data differently and create biases because of their authority over the participants.

N.S.R and S.F.Z are both lecturers. As N.S.R works at the School of Medical Sciences, Universiti Sains Malaysia (SMS USM) and S.F.Z works at the School of Pharmacy, International Medical University (SOP IMU) at the time of the study, they are not affected by their personal perspectives when analyzing the data and thus would not cause potential harm to the participants.

When interpreting the data, all authors could be influenced by their personal experiences as a patient or a relative to a patient, which might negatively affect the participants’ responses.

### 2.7. Ethics

The Ethics Committee for Research Involving Human Subjects, Universiti Putra Malaysia (JKEUPM), granted ethical permission for this study (JKEUPM-2020-415). Before the FGDs and IDIs, all participants received oral and written information about this study, as well as written consent to participate. To protect the participants’ identities, pseudonyms were used during the sessions. Only two identifiable characteristics, namely gender and race, were included in the data display.

## 3. Results

A total of 43 participants took part in the FGDs and IDIs (Table 1). Fifteen physicians took part in FGDs, with the majority of them being Malay and female. In contrast, 17 medical students took part in the FGDs, with the majority being Malay and male. In total, eight FGDs were conducted. Meanwhile, for IDIs, the majority of patients were Chinese and male, as opposed to Malay and female from the relatives.

Pseudonyms that identify the session and the participant’s name were used in quotations to refer to the particular participant. The session for FGDs was indicated by the number 1 to 4. The participant’s name was subsequently represented by the alphabet A through E. For example, Student 1B indicates student B from FGD session one. In the IDIs, the name of the participant was indicated by the number 1 to 6 according to the number of sessions conducted.

This research revealed 44 codes that are categorized into seven themes and 17 categories. Seven essential themes emerged: professionalism, content of communication, verbal, non-verbal, and paraverbal communication skills, environment, and visual communication (Table 2). Quotations are included in this section to support the themes.

### 3.1. Professionalism

Professionalism in the physician-patient relationship was an important component of communication that emerged from the findings, to maintain a good professional relationship between physicians and patients throughout time.

#### 3.1.1. Physician and Patient Attitudes

Participants in FGDs and IDIs emphasized that both physicians and patients should share some of these characteristics including treating each other with politeness, respect, honesty, and being friendly. Most participants also felt that physicians should have empathy toward their patients so that they can treat patients holistically.

*“…. physician can maybe try to be friendly to the patients. So, they would feel more comfortable, and feel like, less worry even though they are diagnosed with a certain disease”*

*[Student 4A]*

*“I think as a patient, we need to be honest with the physician by telling directly to the physician what our past accident was, what happened to us”*

*[Relative 5]*

*“I think that the physician should be able to understand the patient’s situation because we should treat the patient, not treat the disease that the patient presented”*

*[Student 3C]*

#### 3.1.2. Physician Communication Skills

##### Address Patient and Relative Appropriately

Most participants stressed that physicians should address younger patients and relatives by their title and last name. Furthermore, physicians should address the older patients and relatives as ‘uncle’ and ‘aunt’ so that they feel more comfortable and able to put their trust in the physicians.

*“…. maybe the elderly, you can call them uncle and aunty, that’s the starting point. It’s a simple thing because when you gave the proper acknowledgment, we would feel closer to you”*

*[Relative 3]*

##### Use Open-Ended Questions

Participants in this study highlighted that physicians should use open-ended questions during the consultation to obtain as much details as they can from the patients and their relatives. Additionally, patients and relatives would have the chance to express anything they wanted to discuss with their physicians.

*“But generally, we ask open-ended questions (to the patient and relative) to know what’s the complaint”*

*[Physician 3A]*

*“On the aspect of communication, physicians need to have open questions where they welcome us to tell our problems”*

*[Physician 6]*

##### Agreement of Patients’ Privacy and Confidentiality

Medical students explained that physicians must obtain the patient’s consent before informing their families about their illnesses. Some patients seemed uncomfortable involving their relatives, since they do not want to cause them any trouble or distress.

*“…the physicians should really ask the patients if they are comfortable including their relatives in the session as a form of privacy”*

*[Student 1B]*

*“So, sometimes when we ask the patients whether or not their relatives are allowed to be fully involved in managing them, which is also another issue that we have to address”*

*[Student 3A]*

##### Understand Patients’ and Relatives’ Facial Expressions

The participants agreed that physicians should be able to predict how well the patients and their relatives have understood what they have been told by observing their facial expressions. For example, if the physician realized that the patient is unable to comprehend the information based on their facial expression, he or she may repeat the explanation to ensure the patient’s understanding. The physician should then reassure the patient’s understanding by their facial expressions after the explanation.

*“Sometimes we can see from their own face whether they totally understood or they are not clear (with the information). So, if they totally understand, I will just proceed. If the patient looks confused, then I will try to explain again”*

*[Physician 1A]*

*“After the re-explanation all the details, then I asked “Do you know what happened to you just now?”or “Do you know what we are trying to plan?” If the patient is able to answer me, then I understand, I am confident that the patient understood what I’m trying to say….”*

*[Physician 4C]*

##### Promote Patients’ and Relatives’ Comprehension

- Identify the patients’ and relatives’ understanding

In addition, most of the participants repeatedly mentioned the necessity for physicians to assess the patient’s and relatives’ awareness of the patient’s condition. Subsequently, they can determine the level of knowledge that the patient and relatives are at, as well as other important information physicians need to provide.

*“So, usually before I start a discussion, I will ask the patient what he knows about the condition he is having to have a general idea of how much I need to explain to the patient”*

*[Physician 1E]*

- Allow time for comprehension

Furthermore, our participants reported that physicians should give their patients more time to digest the information to make sure they have understood completely, because typically, patients need extra time to recall the inputs given to them by their physicians. When patients are given more time to digest the information, they might be able to assess how much of it they have understood, and what details still need to be discussed with their physicians.

*“If they feel it’s too sudden, like too much of information, inputs, and sudden influx of information, give time for them to go home first to think back”*

*[Physician 3B]*

##### Build Rapport

- Identify the background of the patient and relatives

According to our participants, in order to establish a good rapport with patients and their relatives, physicians should be informed about their backgrounds. This way, physicians can use appropriate visual and verbal communication approaches to deliver the information effectively.

*“…. we need to actually look on the patient first, what his or her background, I mean the education level. Some patients, when we gave too much information, they can’t cope with it”*

*[Student 3D]*

- Smile

Only patients and relatives stated that physicians should smile at them when they consulted with them, particularly when they enter the physician’s office. Some of them reported experiencing anxiety, fear, or nervousness every time they meet their physicians. When the physicians are smiling, they can sense positive energy from the physicians.

*“… the physicians need to always smile. It makes me feel calm”*

*[Patient 4]*

#### 3.1.3. Physician’s Clinical Skill

##### Highly Competent

The majority of the participants believed that physicians should be knowledgeable and highly skilled, particularly in their own specialties so that they can provide the best advice and the greatest care possible to their patients.

*“…. I think before we want to explain everything to patients, we need to have good knowledge regarding every disease that we are treating”*

*[Physician 2D]*

*“Yes, like what people are saying, physicians must be an expert in their field. They (should) know how to treat patients with their skills”*

*[Relative 4]*

##### Obtain a Thorough Patient History

Additionally, this study showed that physicians should be proficient in history taking, since it is one of the most crucial aspects of patient care. Without taking a proper history, a physician will not be able to obtain sufficient and correct information from the patients and their relatives.

*“Then, we need to ask regarding the history basically. Need to explore the details of the patient, the family, and the personal history for us to explore the patient, so that we can come out with a conclusion and a proper diagnosis to treat the patient”*

*[Physician 1B]*

### 3.2. Content of Communication

#### 3.2.1. Coverage

In general, the management and compliance, treatment and side effects, advice and counseling, and prognosis of the disease are the issues that are most frequently discussed between physicians, patients, and/or relatives.

##### Management and Compliance

Physicians and students both emphasized management and compliance. They will explain how they are going to treat the patients and ensure that patients comply with the therapy.

*“… a physician needs to ask the patient after they talked about the illness and management, how about the management? Can the patient tolerate the management?”*

*[Student 3B]*

##### Treatment and Side Effects

Additionally, they emphasized the importance of conveying the treatment plans to patients and relatives. To help patients and relatives understand and make the best decision, they should be provided with various treatment options. Aside from that, physicians can explain what will happen to patients if they choose to skip therapy.

*“….. then, we will try to explain what’s the best and suits the patients from the treatments that we offered”*

*[Physician 1E]*

##### Advice and Counselling

Patients and their relatives, on the other hand, believed that the improvement of the patients was a crucial issue that need to be highlighted. They specifically said that they are eager to hear the finest counseling to make them feel better. They tend to comply with the physician’s advice, such as taking their medications on schedule and following the food intake recommendations.

*“After inserting the angioplasty stent, what are the things that my mother can and can’t do” *

*[Relative 1]*

*“For instance, physicians advised me to cut on Naan bread and only eat half a portion of rice from a plate because my blood sugar was high”*

*[Patient 2]*

##### Prognosis

Moreover, patients and their relatives were concerned with how long it will take for the patient to recover, stating that they always ask their physicians for a faster recovery time.

*“From the patient’s point of view, usually we wanted to know about the progress of the patient”*

*[Patient 1]*

#### 3.2.2. Quantity of Information

One of the critical components of communication is the quantity of information being delivered. Study participants emphasized that in order to provide patients and relatives with high-quality healthcare, essential information should be provided by the informed physicians.

##### Sufficient Information

Participants agreed that physicians should provide an appropriate amount of information that both patients and relatives can comprehend. Excessive information by the physician may be exhaustive to them. A few of the participants pointed out that physicians can provide additional information if a patient or relative is curious to learn more about the illness.

*“… if we dump a huge load of information on that patient, I believe even the patient would feel overwhelmed and end up giving up and just even defaulted the treatment, became non-compliance”*

*[Student 2C]*

### 3.3. Verbal Communication Skill

Verbal communication skill was the third critical component in physician-patient communication. Our findings indicated that physicians are required to be proficient in their listening skills and use the preferred language with simple explanations.

#### 3.3.1. Language

##### Use Preferred Language

The participants highlighted that physicians should be able to converse in Malay or the patient’s mother tongue if they do not understand English. Therefore, physicians are strongly encouraged to use the patient’s preferred language for them to better understand the information.

*“Both of us need to speak the same language. This means that if the patient is fluent in the Malay language, we need to speak in the Malay language. If the patient is fluent in English, we speak in English. We may understand each other, what I’m trying to tell the patient or what the patient tries to tell me”*

*[Physician 1A]*

#### 3.3.2. Terms

##### Use Layman and Simple Terms

Most participants agreed that physicians should minimally or refrain from using technical and complicated medical jargon while communicating with their patients and relatives. Patients and relatives may not understand complicated terms, which will influence their compliance with treatment.

*“….. the terms should not be too complex. Don’t use medical terms that are difficult to be understood”*

*[Relative 6]*

#### 3.3.3. Listening

##### Listen Attentively

All participants agreed that physicians need to listen intently to their patients and relatives. This is essential to show that physicians are concerned about the patient’s condition.

*“…. physician should also be there, willing to listen to the patient’s concerns so they can provide good information for the physician to relief their anxious feeling towards what’s going to happen to them”*

*[Student 4B]*

### 3.4. Non-Verbal Communication Skill

The next theme was non-verbal communication, in which the participants emphasized the importance of eye contact, facial expression, body language, and body movement for effective communication.

#### 3.4.1. Eye Contact

##### Good Eye Contact

Only physicians and students mentioned the importance of eye contact. Based on their experience, patients can understand better when there is good eye contact. Additionally, this also showed that the patients are paying attention to them.

*“A good eye contact is when you can talk to patients and see in their eyes……most of the time you have to spend looking in their eyes, so that everything that you explain, they can feel, they can understand”*

*[Physician 4C]*

#### 3.4.2. Facial Expression

##### Appropriate Facial Expression

It was also discovered that only physicians and students stated that physicians should have proper facial expressions while communicating with patients and relatives. It is important to ensure that patients and relatives perceive that the physicians are aware of the circumstances present.

*“it means (we are) trying to keep warm and nice face so that the patient would be more comfortable to speak with the physician and alert that the physician is giving attention”*

*[Student 1A]*

#### 3.4.3. Body Language

##### Maintain Appropriate Body Language

Most participants elaborated that the physicians must not shift their bodies or turn away from the patients or relatives while attending to them. For example, if the physicians are too focused on their computers, this can make patients or relatives feel uneasy or irritated.

*“…. it will be like several body gestures, including leaning forward and always facing your body toward the patient. You need to practice not looking at the computer too frequently”*

*[Student 2A]*

#### 3.4.4. Body Movement

##### Demonstrate Using Hands or Body Movement

The participants also highlighted that using hands and body movement helps physicians, patients, and relatives to communicate more effectively and make verbal explanations easier. Examples include pointing out the painful location on the body, demonstrating how therapy is carried out, and indicating the medication quantity using hands for illiterate patients.

*“Sometimes we demonstrate the movements (during physiotherapy), the positions and all”*

*[Physician 3D]*

*“You need to take this tablet, two tablets, and eat three times a day. I’ll show you using my fingers”*

*[Physician 1C]*

### 3.5. Paraverbal Communication Skill

The next important theme identified in this study data for physician-patient communication was paraverbal communication skill. This skill refers to how people converse information to other people through voice, intonation, pace, and rhythm. In this study, the physician’s pace and tone of voice were the significant factors to consider when speaking with their patients and relatives.

#### 3.5.1. Tone of Voice

##### Use a Soft Tone

Almost all participants in this study reported that physicians should communicate in a soft tone, so that patients and their relatives will be comfortable talking to them. They also pointed out that patients feel frightened by physicians using a harsh tone, which hinders them from communicating further.

*“I mean, everybody, I’m sure would agree that a soft tone is the tone we should use to communicate”*

*[Physician 2D]*

*“It depends on physicians. Some physicians can patiently and gently explain things to you when you ask questions. On the other hand, if the physician raises his tone, I’ll be terrified”*

*[Patient 2]*

#### 3.5.2. Pace of Voice

##### Maintain a Proper Pace

Participants also believed that physicians should maintain their speed of voice by explaining slowly. This is because if their physicians speak too fast, it will be difficult for the patients and relatives to receive and comprehend the information.

*“…. if I wanted to understand more when the physician is speaking too fast, I will request the physician, “Can you please explain a little bit slow?” It will help me on what to do next. I think speed, it’s very important”*

*[Relative 3]*

### 3.6. Environment

The setting of the clinic or hospital is particularly crucial because physicians require a private space to speak with their patients and relatives. Furthermore, some of the participants emphasized the need for proper distance during consultation between physician, patient, and relative.

#### 3.6.1. Consultation Room Environment

##### Private and Confidential

Only physicians highlighted maintaining a private and confidential environment to protect the patient’s information. This is important to ensure the patient’s details cannot be overheard by others.

*“In the hospital setting, most of the time, the environment will be quite tense. When we want to break bad news, we need to find a suitable situation like for example, a quieter place to protect the patient’s privacy and confidentiality” [Physician 2B]*

*[Physician 2B]*

#### 3.6.2. Consultation Room Layout

##### Appropriate Distance

In addition, the participants reported the need to have an appropriate distance arrangement between the physician, patient, and/or relative. It is because too close of a distance makes patients feel inferior to their physicians. Meanwhile, if the distance is too far, the physicians cannot properly treat their patients.

*“………the distance must be appropriate, not far away. Otherwise, we (physicians) can’t do the examination”*

*[Physician 4C]*

*“The physician should not be too close to me because I lack confidence or feel inferior to let him treat me”*

*[Patient 5]*

### 3.7. Visual Communication

The final theme was visual communication; people share information using visual elements such as images, models, or videos. It helps in verbal explanation, similar to one of the non-verbal skills listed above, like body movement.

#### Use Visual Elements

##### Explain Using External Sources

The participants agreed that it is acceptable for physicians to describe the patient’s condition using diagrams, pictures, web pages, or any other external sources. The patients and relatives admitted that employing external sources will boost their understanding of the physician’s explanations.

*“For me, it is more understandable when the physician draws while explaining about my mom’s operation. I had a clear picture”*

*[Relative 5]*

## 4. Discussion

The participants in the present study provided important components to enhance communication between physicians and patients/relatives. Effective communication between patients, relatives, and physicians can improve the delivery of information and enhance the quality of care.

The skill of physicians to understand the facial expressions of their patients and relatives is one of the factors that could affect the use of appropriate non-verbal and visual communication. Previous studies have shown that facial expressions can correctly predict how well patients and their families are comprehending. The patient’s smile and nodding head indicate that he or she may have grasped the information being conveyed as positive feedback [22]. It is essential to comprehend a patient’s expression, since it helps physicians identify discomfort in a patient. When physicians notice that their patients are in extreme pain, they can immediately assess the condition and act quickly to prevent any undesirable consequences [23]. Furthermore, facial expressions are used by humans to express their inner emotions. In the healthcare context, it helps physicians encounter some expression signals that could not be delivered verbally. For example, the patient may spontaneously lift their eyebrows when something confusing happens. The comprehension levels of patients and relatives should be identified by physicians, because they may not verbally share their doubts or misunderstandings [24].

Additionally, this study revealed that physicians need to be aware of the educational backgrounds of patients and relatives. It helps physicians in deciding whether to use medical jargon and language that can be sufficiently understood. The majority of patients and their relatives preferred physicians to use simple terms. The use of medical jargon, particularly for middle-aged and older patients, was the factor that contributed to unhealthy or inefficient physician-patient communication [5,25].

Moreover, patients in our study noted that if physicians communicate in English, they frequently request their relatives to translate for them. According to a previous study, language barriers could also cause misunderstandings between patients and physicians. As a result, hospitals began using translation services like MediBabble, which contributed to an increase in the cost and duration of treatment sessions [26]. To reduce poor communication that may result in medical errors and health discrepancies, physicians strive to avoid using medical jargon and improve their ability to learn a few different languages, so that they can communicate in the language preferred by patients. In addition, not only in Southeast Asia but even in Western nations like the US, the length of the patient’s visit is extended when physicians and patients converse with one another in two different languages [27,28]. Furthermore, similar to the present findings, elderly patients with low levels of education are more likely to receive care if physicians can speak to them in their mother tongue rather than in English, such as Malay or Chinese [29,30]. Most physicians have high levels of literacy, thus frequently give health information at a level beyond what their patients can grasp. Patients who cannot comprehend health information are unable to follow medical instructions, which prevents them from taking the proper actions and making good decisions. As a consequence, patients do not adhere to treatment regimens or practice proper health-self management, bringing up the risks of patient care errors and eventually, poor health outcomes [31].

Physicians should also exhibit their concerns on patients and relatives, and be more attentive by listening to them. Several studies found that strengthening listening skills is one of the essential components to improve communication. Physicians can better respond to patient inquiries by listening, while at the same time taking note of the patient’s concerns and illness [5,32,33]. Poor listening skills can lead to incorrect assumptions, and physicians may jump to inappropriate conclusions in making a diagnosis and putting the patient’s safety at risk [34].

Our study also revealed that it is also essential for physicians to possess excellent knowledge and skills. Most of the patients and relatives preferred physicians with experience and expertise to care about the patient’s condition and provide good counseling. A previous study proved that a lack of knowledge of the disease and treatment options led to poor communication. Due to this, physicians tend to leave their patients insufficiently informed [3]. The lack of experience and knowledge may also influence the physician’s ability to deliver their knowledge [8]. Possessing an excellent knowledge enables them to obtain an accurate history, administer efficient therapies, and manage patient discomfort [35].

Interestingly, the elderly lay participants in this study expressed their preference to be addressed as ‘uncle’ or ‘aunt’ by the physicians. Younger patients and relatives preferred to be called by their title (Mr/Mrs) and surname. Similarly, physicians also thought that this helps them to win the trust of their patients. These differed from the previous findings, which indicated that elderly patients preferred to be addressed using their title and surname [36,37]. The younger patients, on the other hand, wished to be addressed by their first names [37,38]. The discrepancies may be because, in Malaysian culture, physicians can gain patients’ trust and establish harmonious relationships that are distinct from Western culture. This is confirmed by a previous study done in Malaysia that revealed similarity with this study [39].

Additionally, issues related to patient privacy and confidentiality were only discussed by the professionals, i.e., medical students and physicians. These students indicated that physicians need to obtain patient consent to include additional parties such as their relatives during consultation. Gaining patient consent for confidentiality and privacy is one of the most crucial conditions for effective communication that medical students are learning [9,40]. In contrast to medical students, physicians placed more emphasis on the environment setting, such as having comfortable and private areas to perform a consultation. Moreover, they believed that a conducive room would give assurance that their patients’ information will be kept restricted, as mentioned by Moore et al. (2007) [40]. Even though physicians do not verbally obtain consent from their patients, they must be vigilant that patient’s information might be overheard in an open area [41]. None of the patients or relatives paid close attention to their confidentiality and privacy, which may be due to their unawareness of their rights. Consequently, the protection of a patient’s private information tends to be neglected [42,43]. In addition, patients may lack knowledge about what information should be kept private and what can be revealed to outsiders [44].

Physicians and medical students focused on management plans and the consequences during consultation. In a study by Coulter (2005), physicians also tend to focus more on diagnosis and procedures. The physicians were prone to be more open about treatment and management plans with patients who were more educated and financially stable [45]. Meanwhile, being a layperson, patients and relatives were more concerned about the patient’s progression and advice to heal the condition. As opposed to previous studies, patients wanted to participate in decision-making; therefore, the most crucial thing for them is to understand the numerous treatment options [46,47,48]. Interestingly, the patients in this study were not interested in the management plans as they were unable to comprehend the details. They were adamant that obtaining quality counseling is more important to improve a patient’s condition. If patients believe that their physicians do not provide proper advice, they may request a second opinion from another physician [49,50].

Eye contact was consistently highlighted by physicians and medical students. They stated that when attempting to convey a message, they should establish good eye contact with patients and any present relatives. Previous studies have uncovered evidence to support the value of eye contact. Kluttz et al. [49] and Stepanikova et al. [50] discovered that patients regarded eye contact as important to establish “social engagement” and “social attractiveness” with their physicians. While physicians were too focused on their computers, patients felt that they received a lack of assurance regarding their condition [51]. Other than that, physicians’ eye level indicates their interest in their patients. The patients might realize when the physicians appear disinterested based on their eye level [17].

However, the patients and relatives in our study did not mention the importance of eye contact. On the other hand, they emphasized that physicians need to use a soft tone with an appropriate pace to better comprehend the information. This is supported by Douglas et al. [52] and Khanom et al. [53]. Moreover, most patients reported feeling relaxed and at ease when a physician speaks in a gentle and soft tone, which helps to build a harmonious discussion between them. It is necessary to note, however, that patients and relatives seldom or very rarely encounter physicians who speak harshly with their patients. They also stated that when they were unable to comprehend physicians’ explanations, the physicians would re-explain at a slower pace.

The laymen in our study also claimed that they tend to default on the treatments if the physician seems to have a lack of empathy. They stated that physicians are more approachable when smiling at them, and simultaneously they feel more at ease. Meanwhile, physicians and medical students pointed out that they preferred to have an appropriate facial expression according to the patient’s situation. For example, physicians need to display an expression of concern when they wanted to break bad news to their patients and families [54]. Several studies indicated that when physicians expressed empathy and an appropriate facial expression, patients would be more engaged during the discussion. This subsequently builds a pleasant relationship and might enhance the patient’s recovery [9,55].

The findings of this study revealed essential components in physician-patient communication that may help to improve healthcare. There are several elements that were acknowledged by both professionals and laymen, which reflect their significance and importance in the communication. These critical components that suit Malaysian culture should be emphasized during medical training to improve patient outcome and enhance satisfaction of patients and relatives. Concurrently, we find that patient education on their rights to their privacy and confidentiality is vital in order to prevent the decline of trust and confidence in our healthcare system. Moreover, future studies can be done to improve physician-patient communication, evaluate satisfaction of patients and relatives on healthcare services, and increase their knowledge of medical problems and treatment plans.

### Strengths and Limitations of Study

This study is one of the few studies conducted in Malaysia that focused on physician-patient communication. Several critical components of communication between a physician and a patient were explored in this study. It differs from other studies that highlighted specific communication aspects only, such as the physician’s empathy, patient’s satisfaction and trust, patient’s privacy and confidentiality, as well as verbal and non-verbal communication skills. This study’s recruited patients and relatives were from Peninsular Malaysia and Sabah, which represent almost the whole country. As a result, it is believed that this study gathered valuable data from the points of view and experiences of many patients and relatives.

In the meantime, only medical students and physicians from FMHS and HPUPM were recruited, which might not represent the whole Malaysian professional perspectives. Furthermore, FGDs and IDIs were conducted via online platforms. Network problems were encountered in this study, and several participants were tired out as a result of discussions that were delayed on both parts of the participants and the moderator. As a consequence, it was challenging to control the fruitful data collection process.

## 5. Conclusions

This study demonstrated the essential components that should be brought to light in healthcare settings. The purpose of the study was to gain insight from the perspectives of health professionals, trainees, patients, and relatives to provide good quality services. Important components expressed by the participants were professionalism, content of information, verbal, non-verbal and paraverbal communication skills, environment, and visual communication.

Additionally, it was discovered that Malaysian culture differs from Western culture in terms of how patients and relatives prefer to be called or addressed by physicians in order to develop harmonious relationships with them. This study has highlighted additional concerns where patients and their relatives did not focus on the importance of eye contact, although it is one of the important components required by patients and relatives worldwide. Furthermore, it was found that in Southeast Asia and Western countries, patients and their relatives were unaware of their rights to patient confidentiality and privacy. The results indicated how to teach medical students to call or address patients and relatives in accordance with their preferences, and highlighted the necessity for patients and their relatives to be educated about the value of eye contact and confidentiality protections.

## Figures and Tables

**Table 1:** Demographic details of the participants.

Data Collection Method	Groups of Participants	Gender	Ethnicity
FGD	Medical students	9 M 8 F	9 Mal 5 Chin 3 Ind
	Physicians	7 M 8 F	7 Mal 5 Chin 3 Ind
IDI	Patients	3 M 2 F	2 Mal 3 Chin
	Patients’ relatives	2 M 4 F	4 Mal 2 Ind

FGD: focus group discussion, IDI: in-depth interview, M: Male, F: Female, Mal: Malay, Chin: Chinese, Ind: Indian.

**Table 2:** Critical components of communication in physician-patient relationship.

Theme	Category	Sub-Category	Sub-Sub Category
Professionalism	Physician’s and patient’s attitudes	Friendly Polite Respect Honest Empathy	-
	Physician’s communication skill	Address patient and relative appropriately	-
		Use open-ended questions	-
		Agreement of patients’ privacy and confidentiality	-
		Understand patients’ and relatives’ facial expressions	-
		Promote patients’ and relatives’ comprehension	Identify the patients’ and relatives’ understanding Allow time for comprehension
		Build rapport	Identify the background of the patient and relatives Smile
	Physician’s clinical skill	Highly competent Obtain a thorough patient history	-
Content of communication	Coverage	Management and compliance Treatment and side effects Advice and counselling Prognosis	-
	Quantity of information	Sufficient information	-
Verbal communication skill	Language	Use preferred language	-
	Terms	Use layman and simple terms	-
	Listening skill	Listen attentively	-
Non-verbal communication skill	Eye contact	Good eye contact	-
	Facial expression	Appropriate facial expression	-
	Body language	Maintain appropriate body language	-
	Body movement	Demonstrate using hands or body movement	-
Paraverbal communication skill	Tone of voice	Use a soft tone	-
	Pace of voice	Maintain a proper pace	-
Environment	Consultation room environment	Private and confidential	-
	Consultation room layout	Appropriate distance	-
Visual communication	Use visual elements	Explain using external sources	-

## Data Availability

The data of this study are available from the corresponding author for the reasonable request for future reference.
